# Heterogeneity of respiratory distress syndrome: risk factors and morbidity associated with early and late gestation disease

**DOI:** 10.1186/s12884-016-1085-7

**Published:** 2016-09-27

**Authors:** Azar Mehrabadi, Sarka Lisonkova, K.S. Joseph

**Affiliations:** 1 Department of Obstetrics and Gynaecology, University of British Columbia and the Children’s and Women’s Hospital and Health Centre of British Columbia, Vancouver, BC Canada; 2 Department of Epidemiology, Biostatistics, and Occupational Health, McGill University, Purvis Hall, 1020 Pine Ave. West, Montreal, QC H3A 1A2 Canada; 3 School of Population and Public Health, University of British Columbia, Vancouver, BC Canada

**Keywords:** Respiratory distress syndrome, Respiratory morbidity, Hyaline membrane disease, Preterm, Term newborns, Gestational age, Risk factors

## Abstract

**Background:**

Although respiratory distress syndrome (RDS) is considered a disease of prematurity, there is evidence to suggest heterogeneity between early and late gestation RDS. We examined the epidemiologic features of RDS occurring at early and late gestation.

**Methods:**

We conducted a retrospective cohort study including live births in the United States in 2005–06, with information obtained from the National Center for Health Statistics. Early (<32 weeks) and late gestation RDS (≥39 weeks) were contrasted in terms of risk factors and associations with pregnancy complications, obstetric intervention and co-morbidity. Logistic regression was used to quantify the effects of risk factors, while other associations were quantified descriptively.

**Results:**

There were 27,971 RDS cases, yielding an incidence of 6.4 per 1000 live births. Early and late gestation RDS differed in terms of risk factors, with factors such as multi-fetal gestation more strongly associated with early (adjusted odds ratio [aOR] 11.6, 95 % confidence interval 11.0–12.2) compared with late gestation RDS (aOR 3.66, 95 % confidence interval 2.68–4.98). The morbidity correlates of early and late gestation RDS also differed substantially; neonatal seizures were less strongly associated with early (OR 5.90, 95 % confidence interval 3.67–9.47) compared with late gestation RDS (OR 33.1, 95 % confidence interval 27.2–40.2), while meconium aspiration syndrome was not significantly associated with early gestation RDS (OR 1.87, 95 % confidence interval 0.94–3.72) and very strongly associated with late gestation RDS (OR 39.8, 95 % confidence interval 34.7–45.6).

**Conclusions:**

Differences in risk factors and morbidity correlates of early and late gestation RDS suggest that these entities represent two distinct diseases.

## Background

Respiratory distress syndrome in the newborn (RDS) is a disease caused by the absence or inadequate production of pulmonary surfactant and the related underdevelopment of the lungs [1, 2]. The gestational age-specific incidence of RDS has a bimodal distribution, with a first peak in incidence rates at early gestation and a second peak at late term gestation [3]. Such a bimodal pattern of disease incidence has been traditionally interpreted as suggesting disease heterogeneity. For instance, the bimodal age incidence curve of Hodgkin disease was first described by MacMahon in the 1950s and ascribed to etiologic heterogeneity [4, 5]. Subsequent discoveries, including the detection of Epstein-Barr virus (EBV) DNA in tumour cells, have led to the understanding that the early incidence of Hodgkin disease among young adults is caused by primary infection (non-EBV), while the second peak among older adults is due to loss of immunity to latent infection (mostly EBV) [6, 7]. Similarly, the bimodal gestational age incidence of RDS suggests that RDS occurring at early and late gestation represents two distinct disease entities [3]. Proposed mechanisms for RDS at term include meconium aspiration syndrome [8], diabetes [9], cesarean delivery, in particular cesarean delivery without labour [10–13], delay in onset of respiration [14], birth asphyxia (neonatal encephalopathy) [14], congenital anomalies [8], and rare genetic mutations of surfactant protein [2].

Newborns with RDS at late term gestation comprise a significant and under-recognized subpopulation of RDS infants [15]. In addition, studies have not adequately characterized RDS at late term, even though the condition was documented in 1959 and has been routinely identified in subsequent years [8, 14, 15]. We hypothesized that the peak in the incidence of RDS at late gestation signifies etiologic and other heterogeneity as compared with early gestation RDS. We therefore carried out a study to describe the risk factors, morbidity correlates and other epidemiologic features of RDS occurring at early and late gestation.

## Methods

The study was a retrospective cohort study of live births in the United States in 2005 and 2006. These years were chosen for the study as they represented the most recent period that information on RDS status was collected on the birth certificate. Information on these infants was obtained from the National Center for Health Statistics birth-infant death (period linked) data files, a set of publically available databases maintained by the US Center for Disease Control [16]. These files included routinely collected demographic information, maternal and infant characteristics, and birth outcomes. Data were uniformly coded according to standard specifications and were edited and reviewed to satisfy quality control standards [16]. We restricted our study to live births for whom information was based on the 1989 revision of the US standard certificate of live birth, as this version included information on whether the infant was diagnosed with RDS. RDS was defined as a diagnosis of hyaline membrane disease recorded in the birth certificate. Further, we excluded live births with a gestational age outside the 24 to 43 weeks range. Gestational age at birth was based on the clinical estimate of gestation, which studies demonstrate to be more accurate than gestational age based on the reported last menstrual period [17, 18].

We first determined the incidence pattern of RDS by gestational age using the fetuses-at-risk approach [3]. For these calculations, gestational age-specific incidence rates were estimated by using number of RDS cases at any gestational week in the numerator and the number of fetuses (at risk of birth and RDS) at that gestation in the denominator. Cases of RDS were also categorized into those that occurred at <32 weeks, 32–36 weeks, 37–38 weeks and ≥39 weeks, with the gestational age categories chosen a priori based on the previously described incidence pattern of RDS [3]. The rate of RDS at <32 weeks (cumulative incidence between 24 and 31 weeks) was calculated by dividing the number of RDS cases <32 weeks by the number of fetuses at 24 weeks, while the rate of RDS at ≥39 weeks (cumulative incidence between 39 and 43 weeks) was calculated by dividing the number of RDS cases ≥39 weeks by the number of fetuses at 39 weeks. The primary analysis contrasting early vs late gestation RDS in terms of risk factors and morbidity focused on early gestation RDS (at <32 weeks) and late gestation RDS (at ≥39 weeks).

The fetuses-at-risk approach was used for quantifying the effects of risk factors that were considered to be stable (invariant) through the course of pregnancy from 24 weeks gestation onwards. The fetuses-at-risk approach was preferred for this analysis as risk factors such as older maternal age and chronic hypertension affect fetal growth and birth rates and consequently RDS risk. Gestational age was treated as survival time and took into account the fetal-infant time continuum [19]. Maternal and infant characteristics of interest included maternal age (<20, 20–34, and ≥35 years), pre-existing or gestational diabetes mellitus, chronic hypertension, multi-fetal gestation, smoking status, congenital anomalies and infant sex. Logistic regression was used to obtain unadjusted and adjusted odds ratios (OR) and 95 % confidence intervals (CI) for each risk factor associated with RDS at early and late gestation for this fetuses-at-risk analysis of invariant risk factors.

Other potential factors with a variable time of onset in early or late pregnancy (e.g., preeclampsia, placental abruption) were not included in the fetuses-at-risk analysis as our data source did not contain information on their gestational age of onset (thus precluding a time varying assessment of effects). The relationship between pregnancy complications and obstetric interventions and early versus late gestation RDS was studied among live births. Pregnancy complications included placental abruption, placenta previa, and hypertension in pregnancy, eclampsia, and premature rupture of membranes. Obstetric interventions included labour induction and cesarean delivery. We also examined the morbidity correlates of early vs late gestation RDS among live births by examining the association between RDS and other neonatal morbidity including 5-min Apgar scores (<4, 4–7 and ≥7), small-for-gestational age live birth, neonatal seizures, assisted ventilation <30 min, assisted ventilation ≥30 min, birth asphyxia and meconium aspiration syndrome. Infants born small-for-gestational age were identified based on birth weight-for-gestational age being less than the 10th percentile of the sex-specific United States national reference for fetal growth [20]. Birth asphyxia [21] was defined as a 5-min Apgar score <4, neonatal seizures and receipt of any assisted ventilation. Associations between early (and late) gestation RDS and pregnancy complications, obstetric interventions and other neonatal morbidity, were quantified using ORs and 95 % CIs calculated among live births in each gestational age category. No adjustment was attempted for this descriptive analysis.

Sensitivity analyses examined associations between risk factors, pregnancy complications, obstetric interventions and co-morbidity and RDS occurring at 32–36 weeks and 37–38 weeks. Sensitivity analyses also assessed whether results changed upon adding fetal deaths to the fetuses-at-risk analyses. All analyses were carried out using SAS version 9.3. This study used publicly available data and did not include patient identifiers; ethical review was therefore not sought.

## Results

**Fig. 1:**
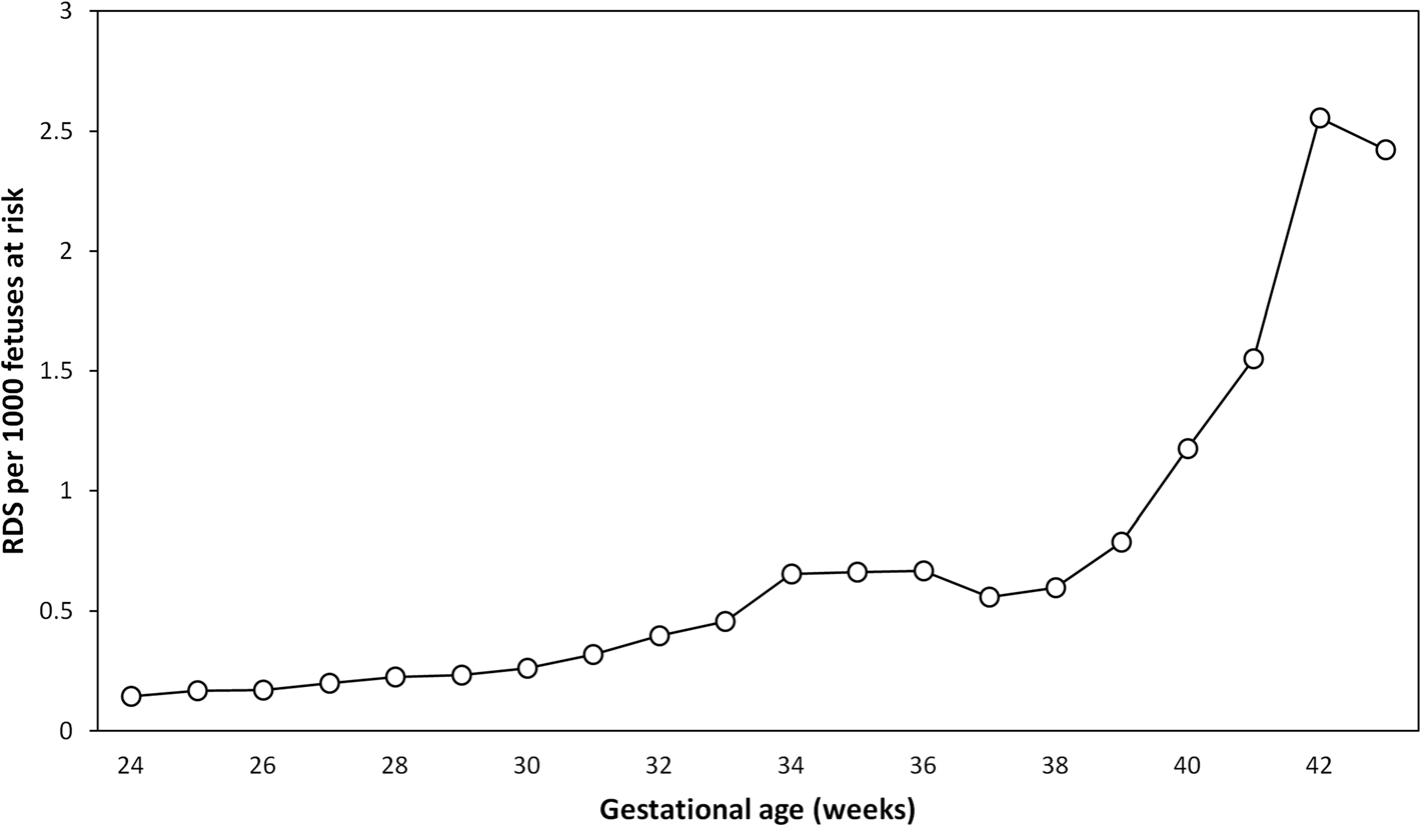
Gestational age-specific incidence pattern of respiratory distress syndrome (RDS) expressed per 1000 fetuses at risk, United States 2005–06

Between 2005 and 2006, a total of 4,368,265 live births contained information on RDS status and the risk factors of interest, and were included in the study. Of these, 27,971 received a diagnosis of RDS, yielding a RDS rate of 6.4 per 1000 live births. Most of the RDS cases occurred at 32–36 weeks gestation (42.7 %); 26.6 % occurred at <32 weeks, 15.3 % occurred at 37–38 weeks and 15.3 % occurred at ≥39 weeks. The cumulative incidence of RDS by gestational week (which approximates the incidence density of RDS) displayed a bimodal incidence pattern by gestational age, peaking at late preterm gestational, then declining slightly at 37 weeks only to rise again at 39 weeks and beyond (Fig. 1).

### Maternal and infant risk factors for early versus late gestation RDS

The cumulative incidence rate of early gestation RDS (between 24 and 31 weeks, denominator of live births and ongoing pregnancies at 24 weeks) was 1.7 per 1000 fetuses at risk, while the cumulative incidence rate of late gestation RDS (between 39 and 43 weeks, denominator of live births and ongoing pregnancies at 39 weeks) was 1.6 per 1000 fetuses at risk. In unadjusted analyses, maternal age <20 years and ≥35 years, multi-fetal gestation, smoking, diabetes, chronic hypertension, small-for-gestational age, congenital anomalies and infant sex were all significantly associated with both early and late gestation RDS (Table 1). Older maternal age (≥35 years) was significantly associated with early gestation RDS but protective for late gestation RDS.

**Table 1:** Rates of respiratory distress syndrome (RDS) per 1000 fetuses at risk at early (<32 weeks) and late (≥39 weeks) gestation and unadjusted odds ratios and 95 % confidence intervals (CI) expressing the association between maternal and infant characteristics and RDS, United States, 2005–2006

Risk factors		RDS <32 weeks				RDS ≥39 weeks			
		Cases	Fetuses at risk	Rate/1000^a^	Odds ratios^b^ [95 % CI]	Casess	Fetuses at risk	Rate/1000^a^	Odds ratios^b^ [95 % CI]
Age	<20 years	959	434,604	2.2	1.38 [1.29, 1.48]	545	272,042	2.0	1.25 [1.14, 1.37]
	20–34	5314	3,312,305	1.6	Reference	3249	2,024,271	1.6	Reference
	≥35	1177	621,356	1.9	1.06 [1.04, 1.08]	493	354,606	1.4	0.95 [0.92, 0.98]
Multi-fetal gestation		2169	150,761	14.4	11.6 [11.1, 12.2]	41	7103	5.8	3.61 [2.65, 4.91]
Smoking		1081	450,149	2.4	1.48 [1.39, 1.58]	582	253,288	2.3	1.49 [1.36, 1.62]
Diabetes		445	163,802	2.7	1.63 [1.48, 1.80]	207	74,377	2.8	1.76 [1.53, 2.02]
Chronic hypertension		355	47,353	7.5	4.60 [4.13, 5.12]	49	17,144	2.9	1.78 [1.34, 2.36]
Congenital anomaly		1000	64,417	15.5	10.5 [9.83, 11.2]	358	31,777	11.3	7.59 [6.81, 8.46]
Male sex		4004	2,235,753	1.8	1.11 [1.06, 1.16]	2581	1,340,148	1.9	1.48 [1.39, 1.57]
All		7450	4,368,265	1.7	-	4287	2,650,919	1.6	-

^a^Rates of RDS <32 weeks represent cumulative incidence from 24 to 31 weeks (8 week period) and cannot be directly compared with rates of RDS ≥39 weeks which represent cumulative incidence from 39 to 43 weeks (5 week period)

^b^Odds ratios were estimated by contrasting the RDS rate among those with and those without the risk factor e.g., RDS < 32 weeks among infants of smokers vs nonsmokers. Since odds ratios express the relative effect of risk factors on RDS, the odds ratios for early gestation RDS can be directly compared with the odds ratios for late gestation RDS

Adjusted analyses showed that several factors differed in their strength of association with early versus late gestation RDS (Table 2). Diabetes mellitus was weakly associated with RDS < 32 weeks (OR 1.31, 95 % CI 1.18–1.44) and more strongly associated with RDS ≥39 weeks (OR 1.74, 95 % CI 1.51–2.00; P value for heterogeneity of odds ratios <0.05). Similarly, male sex was less strongly associated with RDS <32 weeks (OR 1.09, 95 % CI 1.04–1.14) as compared with RDS ≥39 weeks (OR 1.45, 95 % CI 1.36–1.54; P value for difference in odds ratios <0.05). On the other hand, associations between maternal age <20 years, multi-fetal pregnancy, chronic hypertension, and congenital anomalies were stronger with early gestation RDS than with late gestation RDS (Table 2). For instance, chronic hypertension was strongly associated with early gestation RDS (OR 3.89, 95 % CI 3.49–4.35) and moderately associated with late gestation RDS (OR 1.65, 95 % CI 1.25–2.20).

**Table 2:** Adjusted odds ratios and 95 % confidence intervals (CI) expressing the association between maternal and infant characteristics and early (<32 weeks) and late (≥39 weeks) gestation respiratory distress syndrome (RDS), United States, 2005–2006

		Adjusted odds ratios [95 % CI]	
		RDS <32 weeks	RDS ≥39 weeks
Maternal age	<20 years	1.59 [1.48, 1.71]	1.25 [1.14, 1.37]
	20–34	Reference	Reference
	≥35	0.99 [0.97, 1.01]	0.95 [0.92, 0.98]
Multifetal gestation		11.6 [11.0, 12.2]	3.66 [2.68, 4.98]
Smoking		1.53 [1.43, 1.63]	1.44 [1.32, 1.57]
Diabetes		1.31 [1.18, 1.44]	1.74 [1.51, 2.00]
Chronic hypertension		3.89 [3.49, 4.35]	1.65 [1.25, 2.20]
Any congenital anomaly		9.41 [8.79, 10.1]	7.25 [6.50, 8.09]
Male sex		1.09 [1.04, 1.14]	1.45 [1.36, 1.54]

Odds ratios were estimated by contrasting the outcome among those with and those without the risk factor e.g., RDS < 32 weeks among infants of smokers vs non-smokers

### Pregnancy complications and early versus late gestation RDS

The rate of RDS among live births <32 weeks gestation was 113.2 per 1000 live births, whereas the rate among those born at ≥39 weeks was 1.6 per 1000 live births. Rates of early gestation RDS were significantly higher among live births with pregnancy complications such as placental abruption, placenta previa, hypertension in pregnancy, eclampsia and premature rupture of membranes (Table 3). Rates of late gestation RDS were also significantly higher among women with pregnancy complications compared with women without pregnancy complications. However, the associations between pregnancy complications and early versus late gestation RDS were markedly different and substantially stronger between pregnancy complications and late gestation RDS. For instance, placental abruption was moderately associated with early gestation RDS (OR 1.76, 95 % CI 1.63–1.90) and strongly associated with late gestation RDS (OR 6.33, 95 % CI 4.90–8.18; P value for difference in odds ratios <0.05). Similarly, placenta previa, hypertension in pregnancy, eclampsia and premature rupture of membranes were weakly associated with early gestation RDS and strongly associated with late gestation RDS (Table 3).

**Table 3:** Rates respiratory distress syndrome (RDS) at early (<32 weeks) and late (≥39 weeks) gestation per 1000 live births and unadjusted odds ratios and 95 % confidence intervals (CI) expressing the association between pregnancy complications and obstetric interventions, and RDS, United States, 2005–2006

	RDS <32 weeks			RDS ≥39 weeks		
	No.	Rate per 1000	Odds ratio [95 % CI]	No.	Rate per 1000	Odds ratio [95 % CI]
Pregnancy complications						
Placental abruption	836	176	1.76 [1.63, 1.90]	60	10	6.33 [4.90, 8.18]
Placenta previa	199	153.2	1.43 [1.23, 1.67]	25	7.2	4.51 [3.04, 6.69]
Hypertension in pregnancy	1007	154.3	1.50 [1.39, 1.61]	230	3.6	2.31 [2.02, 2.63]
Eclampsia	287	174	1.68 [1.47, 1.91]	27	6.9	4.33 [2.96, 6.33]
Premature rupture of membranes	1510	141.9	1.37 [1.29, 1.46]	107	4.2	2.65 [2.19, 3.21]
Interventions						
Labour induction	280	90.9	0.78 [0.68, 0.88]	1225	1.8	1.17 [1.09, 1.25]
Cesarean delivery	5146	121.9	1.28 [1.22, 1.35]	1881	2.8	2.27 [2.13, 2.41]
Labour induction^a^	115	65.8	0.70 [0.58, 0.85]	947	1.6	1.18 [1.09, 1.27]
Cesarean delivery^a^	2503	94.0	1.12 [1.05, 1.19]	1429	2.3	2.16 [2.02, 2.31]
All live births	7450	113.2	-	4287	1.6	-

^a^Excluding live births with maternal hypertension, eclampsia, diabetes mellitus, placental abruption, placenta previa and any congenital anomaly

### Obstetric interventions and early and late gestation RDS

Table 3 also shows the association between labour induction and cesarean delivery and early versus late gestation RDS. Labour induction had a significant protective effect on early gestation RDS and was a significant but weak risk factor for late gestation RDS. Cesarean delivery was significantly associated with both early and late gestation RDS but the effect was substantially larger at late gestation (OR 1.28 vs OR 2.27; *P* value for difference in odds ratios <0.05). Restricting the analysis to women without pregnancy complications (i.e., without maternal hypertension, eclampsia, diabetes mellitus, placental abruption/previa and congenital anomaly) did not drastically alter the results, although the association between labour induction with early gestation RDS became non-significant.

### Morbidity correlates of early and late gestation RDS

The association between neonatal co-morbidity and early gestation RDS was very different from the association between neonatal co-morbidity and late gestation RDS (Table 4). Apgar score < 4 at 5 min, small-for-gestational age live birth, and meconium aspiration syndrome were not associated with early gestation RDS, while neonatal seizures, ventilation, and birth asphyxia were strongly associated with early gestational RDS. On the other hand, small-for-gestational age live birth was significantly and moderately associated with late gestation RDS, while the other types of neonatal morbidity were all very strongly associated with late gestation RDS. These differences in morbidity correlates between early and late gestation RDs were particularly evident with regard to 5-min Apgar <4, 5-min Apgar 4–6, neonatal seizures, ventilation ≥30 min, birth asphyxia and meconium aspiration syndrome. For example, meconium aspiration syndrome was non-significantly associated with early gestation RDS (OR 1.87, 95 % CI 0.94–3.72) but very strongly associated with late gestation RDS (OR 39.8, 95 % CI 34.7–45.6).

**Table 4:** Rates respiratory distress syndrome (RDS) at early (<32 weeks) and late (≥39 weeks) gestation per 1000 live births and unadjusted odds ratios and 95 % confidence intervals (CI) expressing the association between neonatal co-morbidity and RDS, United States, 2005–2006

		RDS <32 weeks			RDS ≥39 weeks		
		No.	Rate per 1000	Odds ratio [95 % CI]	No.	Rate per 1000	Odds ratio [95 % CI]
Apgar at 5 min	<4	404	111.5	1.03 [0.92, 1.14]	108	45	33.1 [27.2, 40.2]
	4–6	1272	142.1	1.36 [1.27, 1.45]	421	34.1	24.8 [22.4, 27.5]
	≥7	5688	108.8	Reference	3741	1.4	Reference
Small for gestational age		786	113	1.00 [0.92, 1.08]	562	2.6	1.73 [1.58, 1.89]
Neonatal seizures		30	428.6	5.90 [3.67, 9.47]	30	22.6	14.4 [10.0, 20.7]
Ventilation ≥ 30 min		4416	320.9	7.64 [7.26, 8.04]	695	93.6	75.9 [69.7, 82.6]
Ventilation <30 min		503	145.4	1.36 [1.23, 1.50]	351	9.5	6.36 [5.70, 7.10]
Birth asphyxia		4983	255.5	6.20 [5.88, 6.53]	1106	23.5	19.7 [18.4, 21.2]
Meconium aspiration syndrome		10	192.3	1.87 [0.94, 3.72]	228	57.6	39.8 [34.7, 45.6]
All live births		7450	113.2	-	4287	1.6	-

Birth asphyxia was defined as Apgar at 5 min <4, presence of neonatal seizures, or any assisted ventilation. Births with missing Apgar values excluded from the analysis

### Sensitivity analyses

The strength of the association between the factors studied and RDS at 32–36 and 37–38 weeks was generally in between that observed with early gestation RDS (<32 weeks) and late gestation RDS (≥39 weeks) with some exceptions. Diabetes mellitus was more strongly associated with RDS at 37–38 weeks (OR 2.74, 95 % CI 2.74–3.03) compared with RDS at other gestational ages. Similarly, the association between congenital anomalies and RDS at 32–36 weeks gestation (OR 4.47, 95 % CI 4.41–5.09) was stronger than that observed with RDS in other gestational age categories. Associations between placenta previa, eclampsia, premature rupture of membranes and RDS at 37–38 weeks were similar in magnitude to associations between the same complications and RDS at 32–36 weeks, while associations between neonatal co-morbidity and RDS at 37–38 weeks were more similar to associations between neonatal co-morbidity and RDS at ≥39 weeks (Appendix Tables 5–8). Sensitivity analyses including stillbirths in the denominator made no marked change in the results or interpretation (Appendix Tables 9–10).

## Discussion

Our study showed that RDS occurring at late term gestation (≥39 weeks) constituted an important fraction (15.3 %) of RDS. Early and late gestation RDS differed in terms of potential etiologic factors; maternal and infant characteristics such as maternal age <20 years, multi-fetal gestation, chronic hypertension, and congenital anomalies were more strongly associated with early gestation RDS, while factors such as pre-existing diabetes, gestational diabetes and male infant sex were more strongly associated with late gestation RDS. Associations between pregnancy complications, obstetric intervention and neonatal morbidity and early RDS also differed from associations with late gestation RDS. Of particular interest were the differential and very strong associations between late gestation RDS and pregnancy complications (such as placental abruption, placenta previa and eclampsia) and neonatal co-morbidity (especially Apgar <4 at 5 min, neonatal seizures, ventilation ≥30 min, birth asphyxia and meconium aspiration syndrome). These differences suggest substantial heterogeneity in the etiology, pathologic features and clinical presentation of early and late gestation RDS.

Our findings confirm previous reports that RDS at term is associated with [8–12, 14] meconium aspiration syndrome, diabetes, cesarean delivery, birth asphyxia and congenital anomalies [8–12, 14]. In addition, we identified several other risk factors for RDS ≥39 weeks including multi-fetal gestation, chronic hypertension and male infant sex. The strong associations between late gestation RDS and Apgar <4 at 5 min, neonatal seizures, ventilation ≥30 min and birth asphyxia appears to be more consistent with a clinical picture of encephalopathy as opposed to surfactant deficiency or other pulmonary causes. The differential and much stronger associations between pregnancy complications such as placental abruption, placenta previa, and eclampsia and late RDS, suggest that the fetal central nervous system is increasingly susceptible to serious compromise at later gestation. The stronger associations between male sex and small-for-gestational age and late gestation RDS may also indicate a greater susceptibility to encephalopathy [22, 23]. There is a substantial body of animal and human literature showing that the fetal gas exchange and oxygenation is significantly reduced at term gestation and beyond [24–27]. Although uterine artery blood flow increases with gestation, blood flow per unit of fetal weight does not, and arterial partial pressure of oxygen, base excess, oxygen saturation, oxygen content, and arterial partial pressure of carbon dioxide are all reduced at late gestation. The increase in rates of placental complications at late gestation is also congruent with epidemiologic models of stillbirth and perinatal death (which also increase at late gestation) [3].

It is unclear why we observed a negative association between induction and RDS at <32 weeks gestation. One possible explanation is that infants induced at <32 weeks (as opposed to those that delivered spontaneously at this age) were more likely to have received antenatal corticosteroid therapy, which is protective for RDS [28]. The association between cesarean delivery and RDS at ≥39 weeks gestation is consistent with findings from previous studies [10–13] which have proposed mechanisms such as lung immaturity [13], the lack of hormonal processes that initiate and propagate labour and which may be involved in lung function, and the effects of labour and vaginal delivery in clearing lung fluid [29].

The limitations of our study include the absence of information on maternal infection and severity of respiratory distress syndrome. In addition, the information in our data source was obtained through routine abstraction and hence may have been subject to some coding variation and transcription errors. However, these problems are likely to have been non-differential by gestational age and RDS status and hence would have had a limited impact on our findings.

## Conclusions

Our study shows that a significant fraction of RDS occurs at late term gestation. Risk factors such as maternal age, pre-existing and gestational diabetes mellitus, multi-fetal gestation, chronic hypertension and infant sex are differently associated with early and late gestation RDS. Also, the associations between pregnancy complications, and neonatal co-morbidity (that are components of neonatal encephalopathy) are much stronger with late gestation RDS as compared with early gestation RDS. The clinico-epidemiologic picture associated with early and late gestation RDS suggests that these two conditions are distinct disease entities. Further research elucidating the pathogenesis of late gestation RDS will help to clarify the management options for better treating this condition.

## Appendix

**Table 5:** Rates of respiratory distress syndrome (RDS) at 32–36 weeks and 37–38 weeks per 1000 fetuses at risk and unadjusted odds ratios and 95 % confidence intervals (CI) expressing the association between maternal and infant characteristics and RDS, United States, 2005–2006

Risk factors		RDS 32–36 weeks				RDS 37–38 weeks			
		Cases	Fetuses at risk	Rate/1000^a^	Odds ratios^b^ [95 % CI]	Cases	Fetuses at risk	Rate/1000^a^	Odds ratios^b^ [95 % CI]
Age	<20 years	1165	426,327	2.7	1.00 [0.94, 1.06]	360	386,323	0.93	0.83 [0.74, 0.92]
	20–34	8969	3,265,775	2.7	Reference	3364	2,978,793	1.13	Reference
	≥35	1808	610,342	3.0	1.03 [1.01, 1.04]	568	545,748	1.04	0.97 [0.95, 1.00]
Multi-fetal gestation		2770	133,526	20.7	9.61 [9.21, 10.0]	214	55,869	3.83	3.63 [3.16, 4.17]
Smoking		1613	441,604	3.7	1.37 [1.30, 1.44]	632	392,595	1.61	1.55 [1.42, 1.69]
Diabetes mellitus		996	160,801	6.2	2.35 [2.20, 2.51]	414	136,882	3.02	2.95 [2.67, 3.27]
Chronic hypertension		423	44,759	9.5	3.52 [3.19, 3.88]	126	35,036	3.60	3.35 [2.81, 4.01]
Congenital anomaly		832	61,034	13.6	5.26 [4.90, 5.65]	346	50,707	6.82	6.71 [6.01, 7.50]
Male sex		7130	2,200,877	3.2	1.42 [1.37, 1.47]	2739	1,994,128	1.37	1.70 [1.59, 1.81]
All		11,942	4,302,444	2.8	-	4292	3,910,864	1.10	-

^a^Rates of RDS <32 weeks represent cumulative incidence from 24 to 31 weeks (8 week period) and cannot be directly compared with rates of RDS ≥39 weeks which represent cumulative incidence from 39 to 43 weeks (5 week period)

^b^Odds ratios were estimated by contrasting the RDS rate among those with and those without the risk factor e.g., RDS < 32 weeks among infants of smokers vs nonsmokers. Since odds ratios express the relative effect of risk factors on RDS, the odds ratios for early gestation RDS can be directly compared with the odds ratios for late gestation RDS

**Table 6:** Adjusted odds ratios and 95 % confidence intervals (CI) expressing the association between maternal and infant characteristics and respiratory distress syndrome (RDS) at 32–36 weeks and 37–38 weeks, United States, 2005–2006

		Adjusted odds ratios [95 % CI]	
		RDS 32–36 weeks	RDS 37–38 weeks
Maternal age	<20 years	1.14 [1.07, 1.21]	0.86 [0.78, 0.96]
	20–34	Reference	Reference
	≥35	0.97 [0.95, 0.98]	0.95 [0.92, 0.97]
Multi-fetal gestation		9.6 [9.2, 10.0]	3.65 [3.18, 4.20]
Smoking		1.42 [1.35, 1.50]	1.55 [1.42, 1.68]
Diabetes mellitus		2.04 [1.90, 2.18]	2.71 [2.45, 3.01]
Chronic hypertension		2.86 [2.59, 3.16]	2.74 [2.29, 3.28]
Any congenital anomaly		4.81 [4.48, 5.17]	6.22 [5.57, 6.94]
Male sex		1.41 [1.36, 1.46]	1.67 [1.57, 1.77]

Odds ratios were estimated by contrasting the outcome among those with and those without the risk factor e.g., RDS 37–38 weeks among infants/fetuses at risk of smokers vs. non-smokers

**Table 7:** Rates respiratory distress syndrome (RDS) at 32–36 weeks and 37–38 weeks per 1000 live births and unadjusted odds ratios and 95 % confidence intervals (CI) expressing the association between pregnancy complications and obstetric interventions, and RDS at 32–36 weeks and 37–38 weeks, United States, 2005–2006

	RDS 32–36 weeks			RDS 37–38 weeks		
	No.	Rate per 1000	Odds ratio [95 % CI]	No.	Rate per 1000	Odds ratio [95 % CI]
Pregnancy complications						
Placental abruption	645	78.0	2.79 [2.57, 3.03]	102	17.9	5.43 [4.45, 6.62]
Placenta previa	348	63.8	2.20 [1.97, 2.46]	44	9.0	2.67 [1.98, 3.59]
Hypertension in pregnancy	1748	44.1	1.55 [1.47, 1.63]	449	6.8	2.12 [1.92, 2.34]
Eclampsia	321	58.0	1.98 [1.77, 2.22]	43	8.9	2.66 [1.97, 3.59]
Premature rupture of membranes	1430	46.3	1.62 [1.53, 1.71]	104	6.3	1.88 [1.55, 2.29]
Interventions						
Labour induction	1455	26.6	0.85 [0.81, 0.90]	953	3.6	1.08 [1.00, 1.16]
Labour induction^a^	697	20.7	0.80 [0.74, 0.87]	586	2.9	1.07 [0.98, 1.17]
Cesarean delivery	6971	40.7	1.84 [1.77, 1.91]	2340	5.7	2.48 [2.34, 2.64]
Cesarean delivery^a^	3826	32.8	1.64 [1.57, 1.72]	1512	4.5	2.32 [2.16, 2.49]
All live births	11,942	30.5	-	4292	3.4	-

Odds ratios were estimated by contrasting outcomes among those with and those without the risk factor e.g., RDS at 37–38 weeks among infants of women delivering with placental abruption vs. infants of women without placental abruption

^a^Excluding live births with maternal hypertension, eclampsia, diabetes mellitus, placental abruption, placenta previa and any congenital anomaly

**Table 8:** Rates respiratory distress syndrome (RDS) at 32–36 weeks and 37–38 weeks per 1000 live births among infants with other neonatal morbidity, and unadjusted odds ratios and 95 % confidence intervals (CI) expressing the association between neonatal comorbidity and RDS at 32–36 weeks and 37–38 weeks, United States, 2005–2006

		RDS 32–36 weeks			RDS 37–38 weeks		
		No.	Rate per 1000	Odds ratio [95 % CI]	No.	Rate per 1000	Odds ratio [95 % CI]
Apgar at 5 minutes	<4	165	85.2	3.13 [2.67, 3.68]	50	34.5	11.4 [8.6, 15.1]
	4–6	733	99.9	3.73 [3.45, 4.04]	309	45.1	15.0 [13.3, 16.9]
	≥7	11,009	28.9	Reference	3917	3.1	Reference
Small for gestational age		2202	32.7	1.09 [1.04, 1.14]	645	4.8	1.48 [1.36, 1.61]
Neonatal seizures		27	102.3	3.63 [2.44, 5.40]	12	20.1	6.04 [3.41, 10.70]
Ventilation ≥ 30 min		3428	236.1	13.4 [12.8, 14.0]	858	144.4	61.5 [56.8, 66.6]
Ventilation <30 min		766	64.9	2.29 [2.12, 2.47]	294	16.3	5.14 [4.56, 5.79]
Birth asphyxia		4244	153.1	8.37 [8.05, 8.71]	1175	46.3	19.2 [18.0, 20.6]
Meconium aspiration syndrome		38	140.7	5.22 [3.70, 7.36]	66	72.8	23.3 [18.1, 30.0]
All live births		11,942	30.5	-	4292	3.4	-

Birth asphyxia was defined as Apgar at 5 minute <4, presence of neonatal seizures, or any assisted ventilation. Births with missing Apgar values excluded from the analysis. Odds ratios were estimated by contrasting outcomes among those with and those without the risk factor e.g., RDS at 37–38 weeks among infants with and without meconium aspiration syndrome

**Table 9:** Sensitivity analysis including stillbirths: Crude odds ratios and 95 % confidence intervals (CI) expressing the association between early antepartum characteristics and respiratory distress syndrome (RDS), United States, 2005–2006

		Unadjusted odds ratios [95 % CI]			
		RDS < 32 weeks	RDS 32–36 weeks	RDS 37–38 weeks	RDS ≥39 weeks
Maternal age	<20 years	1.38 [1.29, 1.48]	1.00 [0.94, 1.06]	0.83 [0.74, 0.92]	1.25 [1.14, 1.37]
	20–34	Reference	Reference	Reference	Reference
	≥35	1.06 [1.04, 1.08]	1.03 [1.01, 1.04]	0.97 [0.95, 1.00]	0.95 [0.92, 0.98]
Multifetal gestation		11.6 [11.1, 12.2]	9.60 [9.20, 10.0]	3.63 [3.16, 4.17]	3.60 [2.65, 4.91]
Smoking		1.48 [1.38, 1.58]	1.37 [1.30, 1.44]	1.55 [1.42, 1.68]	1.49 [1.36, 1.62]
Diabetes		1.63 [1.48, 1.79]	2.35 [2.20, 2.51]	2.95 [2.66, 3.26]	1.76 [1.53, 2.02]
Chronic hypertension		4.57 [4.10, 5.08]	3.51 [3.18, 3.87]	3.35 [2.81, 4.00]	1.78 [1.34, 2.36]
Any congenital anomaly		10.3 [9.61, 11.0]	5.20 [4.84, 5.58]	6.67 [5.97, 7.45]	7.55 [6.78, 8.42]
Male sex		1.11 [1.06, 1.16]	1.42 [1.37, 1.47]	1.70 [1.59, 1.81]	1.48 [1.39, 1.57]

**Table 10:** Sensitivity analysis including stillbirths: Adjusted odds ratios and 95 % confidence intervals (CI) expressing the association between early antepartum characteristics and respiratory distress syndrome (RDS), United States, 2005–2006

		Adjusted odds ratios [95 % CI]			
		RDS <32 weeks	RDS 32–36 weeks	RDS 37–38 weeks	RDS ≥39 weeks
Maternal age	<20 years	1.59 [1.48, 1.71]	1.14 [1.07, 1.21]	0.86 [0.78, 0.96]	1.25 [1.14, 1.37]
	20–34	Reference	Reference	Reference	Reference
	≥35	0.99 [0.97, 1.01]	0.97 [0.95, 0.98]	0.95 [0.92, 0.97]	0.95 [0.92, 0.98]
Multifetal gestation		11.6 [11.0, 12.2]	9.61 [9.20, 10.0]	3.65 [3.18, 4.20]	3.66 [2.68, 4.98]
Smoking		1.53 [1.43, 1.63]	1.42 [1.35, 1.50]	1.55 [1.42, 1.68]	1.44 [1.32, 1.57]
Diabetes		1.31 [1.18, 1.44]	2.04 [1.90, 2.18]	2.71 [2.45, 3.01]	1.74 [1.51, 2.00]
Chronic hypertension		3.89 [3.49, 4.35]	2.86 [2.59, 3.16]	2.74 [2.29, 3.28]	1.65 [1.25, 2.20]
Any congenital anomaly		9.4 [8.79, 10.1]	4.81 [4.48, 5.17]	6.22 [5.57, 6.94]	7.25 [6.50, 8.09]
Male sex		1.09 [1.04, 1.14]	1.41 [1.36, 1.46]	1.67 [1.57, 1.77]	1.45 [1.36, 1.54]

## Abbreviations

CI: Confidence interval
OR: Odds ratio
RDS: Respiratory distress syndrome
